# Surgical Timing and Survival in Advanced High-Grade Serous Ovarian Cancer in the PARP Inhibitor Era

**DOI:** 10.3390/cancers18020245

**Published:** 2026-01-13

**Authors:** Motoko Kanno, Atsushi Fusegi, Naoki Miyazaki, Risako Ozawa, Sachiho Netsu, Yoichi Aoki, Makiko Omi, Hidetaka Nomura, Mayu Yunokawa, Hiroyuki Kanao

**Affiliations:** 1 Department of Gynecologic Oncology, The Cancer Institute Hospital of Japanese Foundation for Cancer Research, 3-8-31 Ariake, Tokyo 135-8550, Japan; atsushi.fusegi@jfcr.or.jp (A.F.); risako.ozawa@jfcr.or.jp (R.O.); sachiho.netsu@jfcr.or.jp (S.N.); yoichi.aoki@jfcr.or.jp (Y.A.); makiko.omi@jfcr.or.jp (M.O.); hidetaka.nomura@jfcr.or.jp (H.N.); mayu.yunokawa@jfcr.or.jp (M.Y.); hiroyuki.kanao@jfcr.or.jp (H.K.); 2 Department of Clinical Planning and Strategy, The Cancer Institute Hospital of Japanese Foundation for Cancer Research, 3-8-31 Ariake, Tokyo 135-8550, Japan; naoki.miyazaki@jfcr.or.jp; 3 Department of Medical Oncology, The Cancer Institute Hospital of Japanese Foundation for Cancer Research, 3-8-31 Ariake, Tokyo 135-8550, Japan

**Keywords:** *BRCA* Proteins, cytoreduction surgical procedures, high-grade serous ovarian carcinoma, neoadjuvant therapy, ovarian neoplasms, PARP inhibitors, treatment outcome

## Abstract

**Background:** The optimal timing of cytoreductive surgery for advanced high-grade serous carcinoma (HGSC) remains a critical unmet question in the modern era of platinum-based chemotherapy and PARP inhibitor (PARPi) maintenance. To address this gap, we compared outcomes following primary debulking surgery (PDS) versus interval debulking surgery (IDS) in a uniformly treated, contemporary cohort. **Methods:** Patients with FIGO stage IIIB–IVB HGSC treated between 2019 and 2023 were retrospectively analyzed. Baseline tumor burden was assessed using detailed radiologic and laparoscopic evaluations, including both presurgical and intraoperative assessments. Progression-free survival (PFS) and overall survival (OS) were examined using multivariable Cox proportional hazards models and reported as adjusted hazard ratios (aHRs). Subgroup analyses were rigorously conducted according to residual disease status and *BRCA* mutation status. **Results:** Among 221 patients (PDS, *n* = 60; IDS, *n* = 151), the median follow-up was 40 months. In the overall cohort, adjusted PFS and OS did not differ significantly between the PDS and IDS groups (PFS: aHR, 1.15; 95%CI, 0.67–1.98; OS: aHR, 1.24; 95%CI, 0.54–2.83). Outcomes were comparable among patients achieving R0 resection. Notably, *BRCA*-mutated patients demonstrated a substantial survival advantage with PDS (*BRCA*-mutated PFS: aHR, 3.34; 95%CI, 1.06–16.67; OS: aHR, 6.07; 95%CI, 2.13–∞), whereas *BRCA* wild-type patients showed no significant difference between surgical strategies. **Conclusions:** The findings suggest that BRCA-mutated patients may derive a survival benefit from PDS, whereas surgical timing had a limited impact on BRCA wild-type disease. This result underscores the importance of integrating molecular profiling, particularly *BRCA* mutation status, with surgical assessment to guide optimal and personalized treatment strategies in the PARPi era.

## Simple Summary

The best timing of surgery for advanced ovarian cancer remains unclear, especially since new drugs called PARP inhibitors have greatly improved patient outcomes. Traditionally, surgery is performed either before chemotherapy (primary surgery) or after initial chemotherapy (interval surgery), but it is unknown whether one approach is better in the modern treatment era. In this study, we examined patients with advanced high-grade serous ovarian cancer who were treated with current standard therapies, including PARP inhibitors. Overall survival was similar between the two surgical approaches when patient backgrounds were carefully adjusted. However, patients with tumors carrying BRCA mutations showed better survival when surgery was performed first. In contrast, surgical timing had little impact on outcomes in patients without BRCA mutations. These findings suggest that the optimal timing of surgery may depend on the biological features of the tumor. Incorporating genetic information such as BRCA status into surgical planning may help personalize treatment and improve outcomes for patients with advanced ovarian cancer.

## 1. Introduction

High-grade serous carcinoma (HGSC) is the most common and aggressive subtype of advanced ovarian cancer. The recent clinical introduction of poly(ADP-ribose) polymerase inhibitors (PARPis) has fundamentally transformed the therapeutic landscape, providing meaningful and durable improvements in progression-free survival (PFS) across multiple Phase III trials [1,2,3,4]. Despite these pharmacological advances, complete cytoreduction (R0 resection) remains a key prognostic determinant in advanced disease. A recent meta-analysis evaluating surgical outcomes in the PARPi era reaffirmed that R0 resection continues to be associated with favorable survival [5]. Furthermore, our prior study indicated that even among patients with high tumor burden, achieving R0 cytoreduction was associated with improved outcomes under contemporary PARPi-based treatment [6]. Collectively, these findings underscore the enduring importance of optimal surgical management, even in the modern therapeutic setting.

A key unresolved question in this context is the optimal timing of surgery: whether primary debulking surgery (PDS) or neoadjuvant chemotherapy (NAC) followed by interval debulking surgery (NAC-IDS) provides superior clinical benefit. Earlier randomized trials, including EORTC and CHORUS, suggested non-inferiority of IDS compared with PDS [7,8], contributing to the widespread adoption of NAC–IDS strategies [9]. Subsequent prospective trials, such as SUNNY and TRUST, were initiated to reappraise the role of upfront surgery using standardized surgical quality criteria [10,11]. However, as patient enrollment began before the broad implementation of PARPi and the final analysis is not yet available, it remains uncertain whether these findings fully reflect outcomes in the current PARPi era.

Most retrospective comparisons of PDS and IDS have been limited by substantial baseline imbalances in tumor burden—differences that directly influence the likelihood of achieving R0 resection and strongly affect prognosis. Because disease extent has been difficult to harmonize across treatment groups, prior observational studies have been unable to provide a definitive assessment of surgical timing within this new molecular context.

To address these critical gaps, we analyzed a contemporary cohort uniformly treated with PARPi-based strategies, in which potential baseline confounding was mitigated through detailed evaluation and harmonization of tumor burden. By examining survival outcomes in the overall population, evaluating prognostic differences among patients achieving R0 resection, and assessing results stratified by *BRCA* mutation status, this study aimed to clarify the optimal timing of cytoreductive surgery in the context of contemporary PARPi-based treatment.

## 2. Materials and Methods

### 2.1. Study Population

This single-center retrospective cohort study included patients with advanced-stage HGSC of ovarian, fallopian tube, or peritoneal origin (FIGO 2014 stage IIIB–IVB) who initiated primary treatment at our institution between January 2019 and December 2023 [12]. The cohort represents a consecutive series of patients initially treated at our institution during the study period, after applying predefined eligibility criteria. Comprehensive clinical information—including medical history, imaging findings, operative reports (with video review), pathological and laboratory results, chemotherapy records, germline and somatic *BRCA* testing results, and survival outcomes—was retrieved from electronic medical records.

Patients were excluded if they were enrolled in clinical trials, had non-HGSC histology, lacked *BRCA* testing, received fewer than four cycles of platinum-based chemotherapy, were transferred to another institution during first-line treatment, did not undergo cytoreductive surgery, or lacked sufficient pretreatment imaging or operative information to assess intra-abdominal disease extent.

### 2.2. Definitions

Disease stage was assigned according to FIGO 2014 criteria, and histological subtyping followed the WHO 2014 classification system [12,13]. Germline *BRCA* mutations were evaluated using BRACAnalysis^®^ (Myriad Genetics, Salt Lake City, UT, USA), and somatic *BRCA* alterations were assessed with the MyChoice^®^ CDx assay (Myriad Genetics, Salt Lake City, UT, USA).

Patients were categorized using established prognostic thresholds: age (≤60 vs. >60 years), body mass index (BMI < 25 vs. ≥25 kg/m^2^), and serum CA125 (<500 vs. ≥500 U/mL) [14]. Ascites was categorized radiologically as (1) none, (2) confined to either the pelvis or upper abdomen, or (3) involving both regions, following methodologies described by Honda et al. [15] and Nasioudis et al. [16]. Pleural effusion was classified as absent, small, or moderate-to-large on imaging [17]. Venous thromboembolism (VTE) was defined as no thrombus, asymptomatic VTE, or symptomatic VTE [18]. Tumor burden was primarily assessed using the Fagotti score [19], and intra-abdominal lymph node metastasis was recorded as present or absent [20].

Lesions, such as umbilical metastases, direct invasion of the rectal mucosa, inguinal metastases, diaphragmatic apex nodules, and resectable mesenteric lymph nodes—although categorized as FIGO stage IV—were not considered “distant metastases,” as they are typically amenable to surgical removal. For analytical purposes, distant metastasis was defined as a disease that is not surgically resectable.

Rather than relying solely on FIGO stage, detailed lesion distribution and tumor spread patterns were incorporated as confounding variables to capture clinically meaningful differences relevant to surgical decision-making.

### 2.3. Surgery and Chemotherapy

The decision to perform PDS or IDS was made through a multidisciplinary evaluation integrating radiologic findings and diagnostic laparoscopy. Unless contraindicated, diagnostic laparoscopy was routinely performed to objectively assess the extent of intra-abdominal disease using the Fagotti score, and surgical timing was determined based on resectability and clinical condition. At our institution, diagnostic laparoscopy is routinely performed to assess the extent of intra-abdominal disease, unless contraindicated. Accordingly, both patients considered for PDS and those planned for NAC underwent laparoscopic evaluation to determine resectability.

Patients in whom intra-abdominal disease could not be adequately assessed were excluded from the study based on predefined criteria. For individuals proceeding to NAC–IDS, diagnostic laparoscopy allowed tissue biopsy prior to chemotherapy initiation.

Cytoreductive surgery aimed for maximal tumor removal, including multi-organ resection when required. Complete cytoreduction was defined as no visible residual tumor (R0), whereas any macroscopic residual disease was classified as R1.

Chemotherapy consisted of paclitaxel (175 mg/m^2^) plus carboplatin (AUC 6) every 21 days, or docetaxel (75 mg/m^2^) plus carboplatin (AUC 5) every 21 days. Patients receiving NAC underwent 3–6 cycles followed by cytoreductive surgery and postoperative chemotherapy to complete eight total cycles, in accordance with institutional standards and prior literature [7].

Maintenance therapy with PARPi was initiated ≥3 weeks after chemotherapy completion, following Japanese guidelines. Treatment consisted of olaparib, olaparib plus bevacizumab for homologous recombination–deficient (HRD) tumors, or niraparib, depending on the *BRCA*/HRD status [1,2,3]. Patients not eligible for PARPi maintenance therapy were those in whom PARPi was not selected based on multidisciplinary chemotherapy conference discussion, rather than solely on homologous recombination status. Specifically, PARPi was not administered in patients with clinically suboptimal platinum sensitivity or in those who discontinued PARPi due to grade ≥ 3 treatment-related adverse events, and bevacizumab maintenance therapy was used according to institutional practice [21].

### 2.4. Endpoints

The primary endpoint was PFS, defined as the interval from the initiation of primary treatment—either PDS or the start of NAC—to the first occurrence of radiologic or clinical disease progression or death from any cause, whichever occurred first. Disease progression was assessed according to RECIST version 1.1, or by clinical judgment in cases of symptomatic deterioration or sustained tumor marker elevation suggestive of progression [22].

The secondary endpoint was overall survival (OS), defined as the time from treatment initiation to death from any cause; patients alive at last follow-up were censored.

Prespecified subgroup analyses evaluated PFS and OS according to (1) residual disease status at cytoreductive surgery (R0 vs. R1) and (2) *BRCA* mutation status (mutated vs. wild-type status), to explore whether the prognostic impact of surgical timing differed according to surgical completeness or underlying molecular background.

### 2.5. Statistical Analysis

Continuous variables were summarized using medians and interquartile ranges (IQRs), while categorical variables were reported as frequencies and percentages.

PFS and OS, including median survival times, were estimated using unadjusted Kaplan–Meier analyses. Survival differences between groups were evaluated using Cox proportional hazards models adjusted for potential confounders. Both crude and adjusted hazard ratios (aHRs) with corresponding 95% confidence intervals (CIs) were calculated [23,24].

In this study, we presented adjusted survival curves derived from the Cox models rather than unadjusted Kaplan–Meier curves. Traditional Kaplan–Meier estimates do not account for imbalances in covariates and may therefore yield biased comparisons between treatment groups. In contrast, adjusted survival curves incorporate covariates into the estimation process, providing more accurate and clinically meaningful comparisons between groups [25].

For analyses involving *BRCA*-mutated patients, the Firth penalized likelihood method was applied to address sparse-event bias, as the number of events—particularly in the PDS group—remained limited even after extended follow-up [26].

Confounders were selected based on well-established prognostic factors in advanced ovarian cancer and included age, BMI, CA125 level, performance status (PS), pleural effusion, ascites, VTE, intra-abdominal tumor extent, distant metastases, and lymph node involvement. Each variable was categorized and analyzed as defined in the Definitions section. Notably, tumor extent was evaluated using detailed, site-specific criteria rather than FIGO stage to capture clinically meaningful differences relevant to surgical decision-making.

Missing data were not imputed. All statistical analyses were performed using EZR version 1.62 (R Software, Vienna, Austria) [27].

### 2.6. Ethics

This retrospective study was conducted in accordance with the Declaration of Helsinki and was approved by the institutional review board of the Cancer Institute Hospital of the Japanese Foundation for Cancer Research (approval number: 2023-GB-066). Given the retrospective nature of the study, the requirement for written informed consent was waived by the institutional review board, and an opt-out consent procedure was implemented in accordance with institutional policy and national ethical guidelines. Information regarding the study was disclosed publicly, and patients were provided with the opportunity to refuse participation. STROBE guidelines were followed. In accordance with the journal’s guidelines, the data will be made available for independent analysis by a team selected by the Editorial Office if requested.

## 3. Results

### 3.1. Patient Characteristics

A total of 331 patients with FIGO stage IIIB–IVB epithelial ovarian cancer were treated at our institution during the study period, of whom 280 were diagnosed with HGSC. The numbers of patients excluded for each predefined criterion are detailed in Figure S1. After applying the eligibility criteria, 221 patients were included in the analysis: 60 in the PDS group and 151 in the IDS group (Figure S1). The median follow-up period was 40 (range, 11–85) months. *BRCA* mutations were identified in 18 patients (30%) in the PDS group and 54 (35.8%) in the IDS group. Complete resection (R0) was achieved in 78.3% of the PDS group and 72.8% of the IDS group (Table 1).

### 3.2. Survival Analysis Comparing PDS and IDS in the Overall Cohort

**Figure 1:**
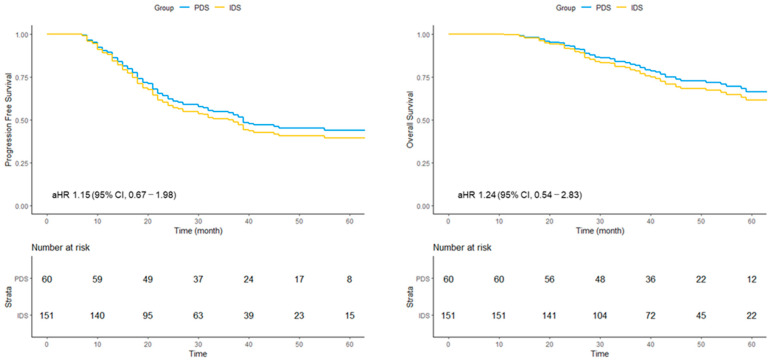
Adjusted survival curves for progression-free survival (PFS) and overall survival (OS) in the overall cohort undergoing primary or interval debulking surgery. Survival curves were derived from multivariable Cox proportional hazards models. IDS, interval debulking surgery; PDS, primary debulking surgery; aHR, adjusted hazard ratio; CI, confidence interval.

In the overall cohort, the median PFS was 65 (range, 38–not reached) months in the PDS group and 25 (range, 22–38) months in the IDS group. The crude HR (cHR) for PFS was 1.84 (95% CI, 1.18–2.87), whereas the adjusted HR (aHR) was 1.15 (95% CI, 0.67–1.98). For OS, the median was not reached in either group. The cHR was 2.30 (95% CI, 1.17–4.54), and the aHR was 1.24 (95% CI, 0.54–2.83) (Figure 1).

### 3.3. Survival Analysis Stratified by Residual Disease Status

Among patients who achieved R0 resection, the median PFS was 80 (range, 30–not reached) months in the PDS group and 37 (range, 24–not reached) months in the IDS group. Among those with R1 disease, the median PFS was 39 (range, 14–not reached) months for PDS and 18 (range, 13–23 months) months for IDS. 

Using PDS/R0 as the reference, the aHRs for PFS were 1.22 (95% CI, 0.63–2.37) for IDS/R0, 1.53 (95% CI, 0.56–4.14) for PDS/R1, and 2.05 (95% CI, 0.96–4.36) for IDS/R1.

**Figure 2:**
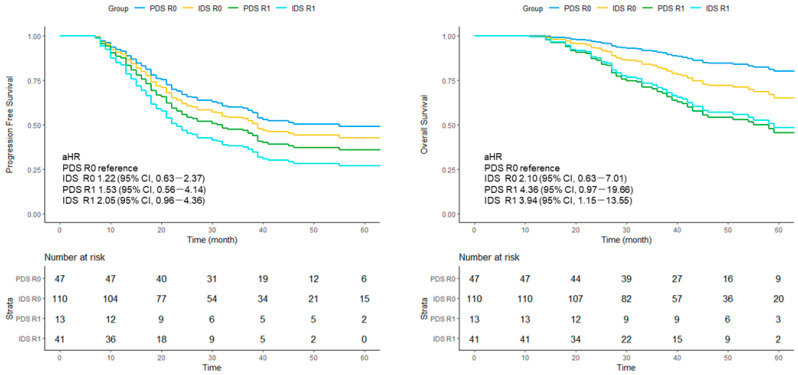
Adjusted survival curves for progression-free survival (PFS) and overall survival (OS) stratified by residual disease status following primary or interval debulking surgery. Survival curves were generated from multivariable Cox proportional hazards models adjusting for predefined clinical and disease-related covariates. PDS, primary debulking surgery; IDS, interval debulking surgery; aHR, adjusted hazard ratio. R0: no macroscopic residual disease. R1: macroscopic residual disease.

A similar pattern was observed for OS. Compared with PDS/R0, the aHRs were 2.10 (95% CI, 0.63–7.01) for IDS/R0, 4.36 (95% CI, 0.97–19.66) for PDS/R1, and 3.94 (95% CI, 1.15–13.55) for IDS/R1 (Figure 2).

### 3.4. Survival Analysis Stratified by BRCA Mutation Status

#### 3.4.1. BRCA-Mutated Subgroup

**Figure 3:**
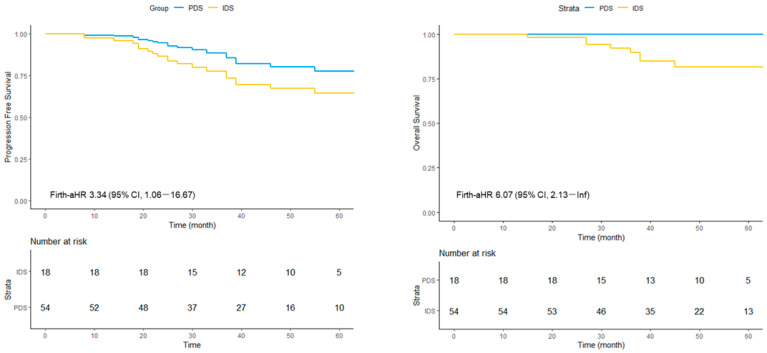
Adjusted survival curves for progression-free survival (PFS) and overall survival (OS) in patients with BRCA-mutated tumors undergoing primary or interval debulking surgery. Survival curves were derived from Cox proportional hazards models using Firth’s penalized likelihood method to address sparse-event bias. The wide confidence intervals reflect limited event numbers in this subgroup. IDS, interval debulking surgery; PDS, primary debulking surgery; aHR, adjusted hazard ratio; CI, confidence interval.

Among patients with mutated *BRCA*, the median PFS was not reached in either treatment group. The Firth-aHR for PFS comparing IDS with PDS was 3.34 (95% CI, 1.06–16.67). Similarly, the median OS was not reached in either group, while the corresponding Firth-adjusted HR was 6.07 (95% CI, 2.13–Inf) (Figure 3). The adjusted hazard ratios were associated with wide confidence intervals, indicating substantial statistical uncertainty, particularly in subgroup analyses with a limited number of events.

#### 3.4.2. BRCA Wild-Type Subgroup

**Figure 4:**
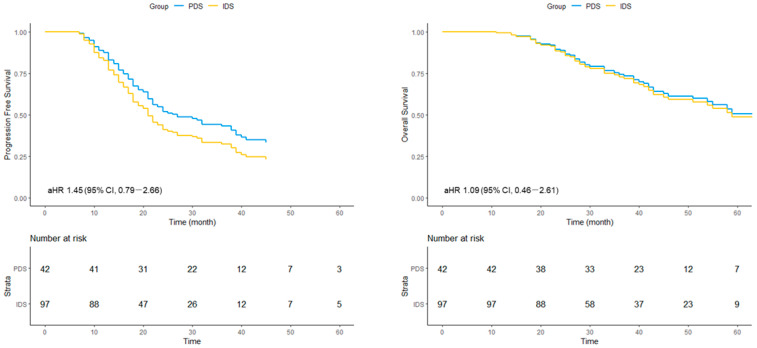
Adjusted survival curves for progression-free survival (PFS) and overall survival (OS) in patients with BRCA wild-type tumors undergoing primary or interval debulking surgery. Survival curves represent covariate-adjusted estimates derived from multivariable Cox proportional hazards models. IDS, interval debulking surgery; PDS, primary debulking surgery; aHR, adjusted hazard ratio; CI, confidence interval.

In patients with wild-type *BRCA*, the median PFS was 38 (range, 22–not reached) months in the PDS group and 19 (range, 17–22) months in the IDS group. The cHR was 1.93 (95% CI, 1.20–3.09), and the aHR was 1.45 (95% CI, 0.79–2.66). For OS, the median was 65 months in the PDS group and 54 months in the IDS group. The cHR was 2.23 (95% CI, 1.12–4.44), and the aHR was 1.09 (95% CI, 0.46–2.61) (Figure 4).

Finally, in an exploratory analysis restricted to *BRCA* wild-type patients achieving R0 resection, no significant differences were observed between PDS and IDS. The aHR for PFS was 1.20 (95% CI, 0.53–2.69), and the aHR for OS was 0.87 (95% CI, 0.17–4.29), with the median OS not reached in either group (Figures S2 and S3).

## 4. Discussion

This study re-evaluated the prognostic relevance of surgical timing in advanced HGSC by analyzing a contemporary cohort uniformly treated in the PARPi era and by harmonizing baseline tumor burden—an important methodological improvement over prior retrospective comparisons [28,29]. After multivariable adjustment for detailed clinical and disease-related factors, survival outcomes did not differ significantly between PDS and IDS in the overall population, with aHRs close to unity. Similarly, among patients achieving complete cytoreduction (R0), PFS and OS were comparable between the two surgical strategies. These findings suggest that when R0 resection is achieved within a uniformly treated contemporary cohort, the independent prognostic contribution of surgical timing may be limited.

Following the publication of the EORTC and CHORUS trials, the use of neoadjuvant chemotherapy followed by interval debulking surgery (NAC–IDS) has increased substantially in real-world clinical practice. Population-based analyses have demonstrated a marked rise in NAC–IDS utilization over time, with consistent trends observed across patients with stage III–IV disease [9].

Importantly, the present findings should not be interpreted as supporting the routine preference of NAC–IDS solely on the basis of comparable survival outcomes. Although NAC–IDS has been associated with lower perioperative morbidity in previous studies, survival equivalence alone does not justify the indiscriminate selection of NAC–IDS in patients who are considered resectable upfront.

At our institution, surgical timing is determined through multidisciplinary evaluation, and patients judged to be suitable candidates for primary debulking surgery (PDS) are preferentially treated with upfront surgery. Therefore, the present results reflect outcomes under a strategy that actively prioritizes PDS when complete cytoreduction is considered achievable.

A central finding of this study is the marked heterogeneity in the prognostic impact of surgical timing according to the *BRCA* mutation status. Among patients with *BRCA*-mutated tumors, PDS was associated with favorable survival outcomes, which remained significant after rigorous multivariable adjustment using Firth’s penalized likelihood method. In contrast, surgical timing appeared to have minimal prognostic influence among *BRCA* wild-type patients, both in the overall cohort and in analyses restricted to those achieving R0 resection. These results indicate that the effect of surgical timing in advanced HGSC is not uniform but is substantially modified by underlying tumor biology.

This observation warrants consideration in the context of HRD. Comprehensive HRD status could not be incorporated as a primary covariate due to substantial missingness following its mid-study introduction; however, descriptive HRD data are provided in the Supplementary Materials. Exploratory analyses did not demonstrate a clear differential effect of surgical timing within the broader HR-deficient subgroup, although these findings should be interpreted cautiously given incomplete data and limited statistical power. Collectively, our results suggest that the observed benefit of PDS may be more pronounced in *BRCA*-mutated tumors, which represent the most profound form of HRD and are characterized by marked platinum sensitivity and exceptional vulnerability to PARPi [30]. Maximal upfront cytoreduction in this biological context may exert an amplified therapeutic effect. Early removal of macroscopic disease could reduce the reservoir of PARPi-resistant subclones, thereby enhancing the durability of subsequent maintenance therapy [31]. Our findings extend earlier reports of a PDS benefit in BRCA-mutated patients [32] into a contemporary setting in which PARPi maintenance was routinely administered.

In contrast, outcomes among *BRCA* wild-type tumors were more heterogeneous. These tumors exhibit greater biological diversity and are less consistently sensitive to platinum-based chemotherapy and PARPi [33]. As a result, recurrence risk in *BRCA* wild-type disease may be driven more substantially by factors beyond macroscopic cytoreduction alone. In particular, microscopic residual disease—undetectable despite achieving macroscopic R0—may contribute meaningfully to recurrence dynamics, potentially diminishing any incremental benefit of upfront cytoreduction [34]. The absence of a clear PDS benefit in this subgroup underscores the importance of incorporating tumor biology, in addition to resectability, into surgical decision-making.

The numerically worse unadjusted outcomes observed in the IDS group were largely attenuated after multivariable adjustment, suggesting that baseline differences in tumor burden remained an important confounder despite efforts at harmonization. In addition, potential biological effects of NAC, including the selection or expansion of platinum-resistant subclones, have been proposed in prior studies [35,36,37]. While causal relationships cannot be established in this retrospective analysis, these considerations highlight the need for further investigation of NAC-associated biological effects, particularly within molecularly defined subgroups. Importantly, NAC–IDS remains an essential strategy for patients with initially unresectable disease or limited physiological reserve.

Recent conceptual frameworks have emphasized that selection between PDS and IDS should consider not only tumor resectability, but also institutional surgical expertise and tumor biology [38]. Our findings should be interpreted alongside the TRUST trial, which highlighted the potential benefit of PDS when high surgical quality was strictly mandated [11]. Together, these data suggest that the impact of surgical timing may reflect an interaction between surgical quality and intrinsic tumor biology, particularly in the PARPi era. While our results do not challenge the established role of NAC–IDS in appropriately selected patients, they highlight the potential value of incorporating molecular characteristics—most notably *BRCA* mutation status—into individualized surgical planning.

This study has several strengths. We focused exclusively on HGSC, incorporated granular assessments of tumor burden beyond FIGO staging, and evaluated patients treated under a uniform PARPi-based maintenance strategy at a single high-volume institution. Therefore, surgical judgment and operative technique were consistent, and multivariable analyses included a wide range of validated clinical covariates. In addition, the application of Firth’s penalized likelihood method allowed for more stable risk estimation in subgroups with limited event numbers, particularly among *BRCA*-mutated patients.

However, several limitations should be acknowledged. In this study, complete cytoreduction (R0) was defined based on intraoperative macroscopic assessment by the operating surgeon, and postoperative imaging was not routinely performed to confirm the absence of residual disease. Therefore, the presence of occult residual disease cannot be excluded even among patients classified as having achieved R0 resection, which may have influenced survival outcomes. The retrospective, single-center design limits generalizability, and the median follow-up of 40 months, while adequate for medium-term outcomes, may be insufficient to fully capture late recurrences—especially among *BRCA*-mutated patients receiving long-term PARPi maintenance. In addition, maintenance therapy consisted of different PARPi regimens, including olaparib monotherapy, olaparib plus bevacizumab, and niraparib, and the potential differential impact of PARPi type on survival outcomes could not be fully assessed. However, treatment selection was determined through a standardized multidisciplinary chemotherapy conference at our institution, and systematic differences in PARPi use between the PDS and IDS groups were minimized. Nevertheless, residual confounding related to surgical judgment, patient frailty, and unmeasured biological factors cannot be completely excluded, even with a standardized institutional decision-making process. Subgroup analyses, particularly in BRCA-mutated patients undergoing PDS, were constrained by small sample sizes and should therefore be interpreted with caution. In addition, the number of events—particularly in the PDS group and in BRCA-mutated subgroups—was limited, resulting in wide confidence intervals for several adjusted hazard ratio estimates. These findings should therefore be interpreted with caution and regarded as hypothesis-generating rather than definitive. Although Firth’s penalized likelihood method was applied to reduce small-sample bias in analyses with sparse events, it does not overcome the fundamental limitation imposed by low event counts. Finally, microscopic peritoneal disease, which may persist despite macroscopic R0 resection, was not directly measurable and may influence recurrence patterns, especially *BRCA* wild-type disease.

Despite these limitations, our study provides clinically relevant insight into surgical decision-making for advanced HGSC in the PARPi era. While adjusted survival outcomes were comparable between PDS and IDS overall, *BRCA*-mutated patients demonstrated a potential survival advantage with PDS, whereas surgical timing had a limited prognostic impact in *BRCA* wild-type disease. These findings underscore the importance of integrating molecular profiling with individualized assessments of resectability and institutional surgical capacity when selecting the optimal cytoreductive strategy. Prospective studies stratified by *BRCA* and HRD status are warranted to validate these observations and to further refine personalized treatment approaches in advanced HGSC.

## 5. Conclusions

In advanced high-grade serous ovarian carcinoma treated in the PARPi era, survival did not differ between PDS and IDS after adjustment, including among patients achieving R0 resection. *BRCA*-mutated patients appeared to benefit more from PDS, whereas surgical timing had a limited impact on *BRCA* wild-type disease. Integrating molecular status with resectability assessment is essential for optimizing surgical strategy. These findings suggest that the prognostic impact of surgical timing in advanced HGSC may be modified by underlying tumor biology, particularly BRCA mutation status, in the PARPi era. Importantly, comparable survival outcomes between PDS and NAC–IDS should not be interpreted as supporting the routine preference of NAC–IDS in patients who are considered resectable upfront. While NAC–IDS remains an essential strategy for patients with initially unresectable disease or limited physiological reserve, careful patient selection integrating molecular characteristics, resectability assessment, and institutional surgical expertise is critical. Prospective studies stratified by BRCA and homologous recombination status are warranted to further refine personalized surgical strategies.

## Figures and Tables

**Table 1:** Baseline characteristics of patients undergoing primary or interval debulking surgery.

Factor	Group	PDS (*n* = 60)	IDS (*n* = 151)
Age, years, median (IQR)		55.00 [50.00, 63.25]	55.00 [49.00, 66.00]
Age group, *n* (%)	<60 years	38 (63.3)	90 (59.6)
	≥60 years	22 (36.7)	61 (40.4)
Performance status, *n* (%)	0	57 (95.0)	101 (66.9)
	1	3 (5.0)	44 (29.1)
	2	0 (0.0)	6 (4.0)
BMI, kg/m^2^, median (IQR)		21.30 [19.27, 23.14]	20.87 [19.23, 23.78]
BMI group, *n* (%)	<25	51 (85.0)	129 (85.4)
	≥25	9 (15.0)	22 (14.6)
CA125, U/mL, median (IQR)		596.10 [116.05, 1717.30]	1912.40 [772.75, 4194.00]
CA125 group, *n* (%)	<500	27 (45.0)	19 (12.6)
	≥500	33 (55.0)	132 (87.4)
VTE, *n* (%)	None	57 (95.0)	127 (84.1)
	Asymptomatic	2 (3.3)	22 (14.6)
	Symptomatic	1 (1.7)	2 (1.3)
FIGO stage, *n* (%)	IIIB–IIIC	47 (78.3)	56 (37.1)
	IVA–IVB	13 (21.7)	95 (62.9)
Ascites, *n* (%)	None	40 (66.7)	53 (35.1)
	Pelvis or upper abdomen	13 (21.7)	37 (24.5)
	Pelvis and upper abdomen	7 (11.7)	61 (40.4)
Pleural effusion, *n* (%)	None	57 (95.0)	104 (68.9)
	Small	2 (3.3)	32 (21.2)
	Moderate to large	1 (1.7)	15 (9.9)
Intra-abdominal lymph node metastasis ^†^, *n* (%)	None	37 (61.7)	65 (43.0)
	Present	23 (38.3)	86 (57.0)
Distant metastasis *, *n* (%)	None	53 (88.3)	82 (59.4)
	Present	7 (11.7)	56 (40.6)
Fagotti score, *n* (%)	0	5 (8.3)	2 (1.3)
	2	8 (13.3)	4 (2.6)
	4	22 (36.7)	10 (6.6)
	6	20 (33.3)	56 (37.1)
	8	2 (3.3)	40 (26.5)
	10	2 (3.3)	25 (16.6)
	12	1 (1.7)	13 (8.6)
	14	0 (0.0)	1 (0.7)
*BRCA* status, *n* (%)	Mutant	18 (30.0)	54 (35.8)
	Wild type	42 (70.0)	97 (64.2)
HRD status, *n* (%)	HR deficient	28 (46.7)	74 (49.0)
	HR proficient	13 (21.7)	40 (26.5)
	No data	19 (31.7)	37 (24.5)
PARPi maintenance, *n* (%)	None	11 (18.3)	30 (20.0)
	Received	49 (81.7)	120 (80.0)
Residual disease, *n* (%)	R0 ^†^	47 (78.3)	110 (72.8)
	R1 ^‡^	13 (21.7)	41 (27.2)

IQR, interquartile range; BMI, body mass index; CA125, carbohydrate antigen 125; VTE, venous thromboembolism; FIGO, International Federation of Gynecology and Obstetrics; *BRCA*, breast cancer gene; HRD, Homologous Recombination Deficiency; PARPi, Poly (ADP-ribose) polymerase inhibitors; PDS, Primary debulking surgery; IDS, Interval debulking surgery. * Distant metastasis: Umbilical metastases, direct invasion of the rectal mucosa, and inguinal, cardiophrenic angle, and some resectable mesenteric lymph nodes were excluded. ^†^ R0: no macroscopic residual disease. ^‡^ R1: macroscopic residual disease.

## Data Availability

The data presented in this study are available on request from the corresponding author.

## Supplementary Materials

The following supporting information can be downloaded at https://www.mdpi.com/article/10.3390/cancers18020245/s1. Figure S1: Consort diagram demonstrating patient flow; Figure S2: Adjusted survival curves for primary and interval debulking surgery among BRCA wild-type patients who achieved complete cytoreduction (R0); Figure S3: Adjusted survival curves for primary and interval debulking surgery among patients with homologous recombination–deficient (HRD) tumors.

## Abbreviations

The following abbreviations are used in this manuscript:

AUC	Area Under the Curve
aHR	Adjusted Hazard Ratio
BMI	Body Mass Index
BRCA	Breast Cancer Gene
cHR	Crude Hazard Ratio
FIGO	International Federation of Gynecology and Obstetrics
HGSC	High-Grade Serous Carcinoma
HRD	Homologous Recombination Deficiency
IDS	Interval Debulking Surgery
NAC	Neoadjuvant Chemotherapy
OS	Overall Survival
PARPi	Poly(ADP-ribose) Polymerase Inhibitor
PDS	Primary Debulking Surgery
PFS	Progression-Free Survival
PS	Performance Status
RECISTs	Response Evaluation Criteria in Solid Tumors
R0	Complete Cytoreduction (no visible residual tumor)
R1	Macroscopic Residual Disease
VTE	Venous Thromboembolism
WHO	World Health Organization
