# Supplementary material for: Surgical Timing and Survival in Advanced High-Grade Serous Ovarian Cancer in the PARP Inhibitor Era
Source: Cancers (Basel). 2026 Jan 13;18(2):245. doi: 10.3390/cancers18020245 (PMC12839418; doi:10.3390/cancers18020245)
Supplement: Supplementary file 1 [file cancers-18-00245-s001.zip › cancers-4084829-supplementary.pdf]

cancers

MDPI

supplementary materials

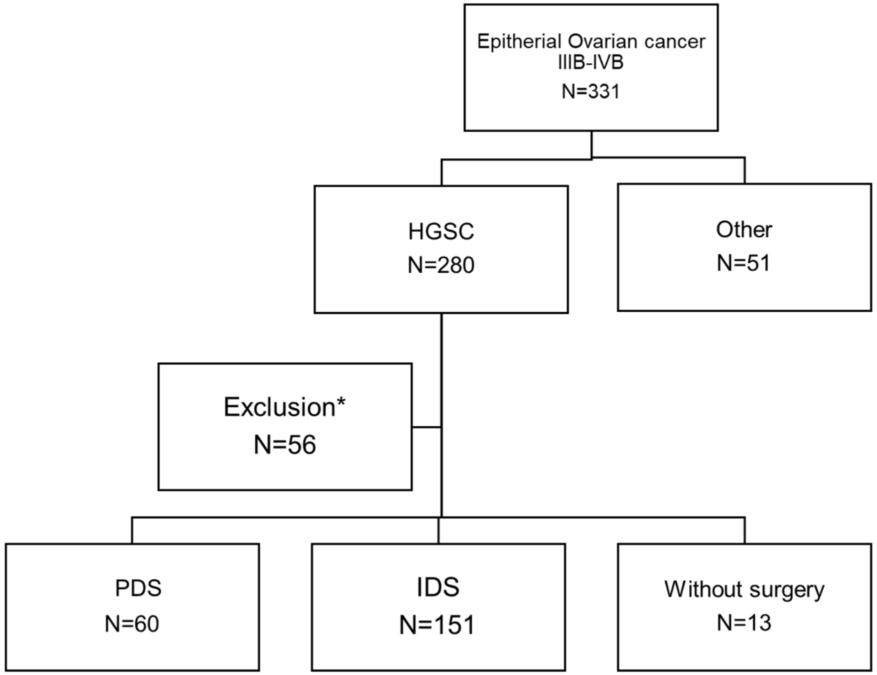

Figure S1. Consort diagram demonstrating patient flow. HGSC, high grade serous carcinoma; PDS, primary debulking surgery; IDS, interval debulking surgery. *Exclusion: Clinical trial=14, only surgical treatment (Hospital transfer)=25, Inadequate chemotherapy=2, Unknown *BRCA* gene mutation=4, Unknown intra-abdominal status prior to treatment=24

 https://doi.org/10.3390/cancers18020245

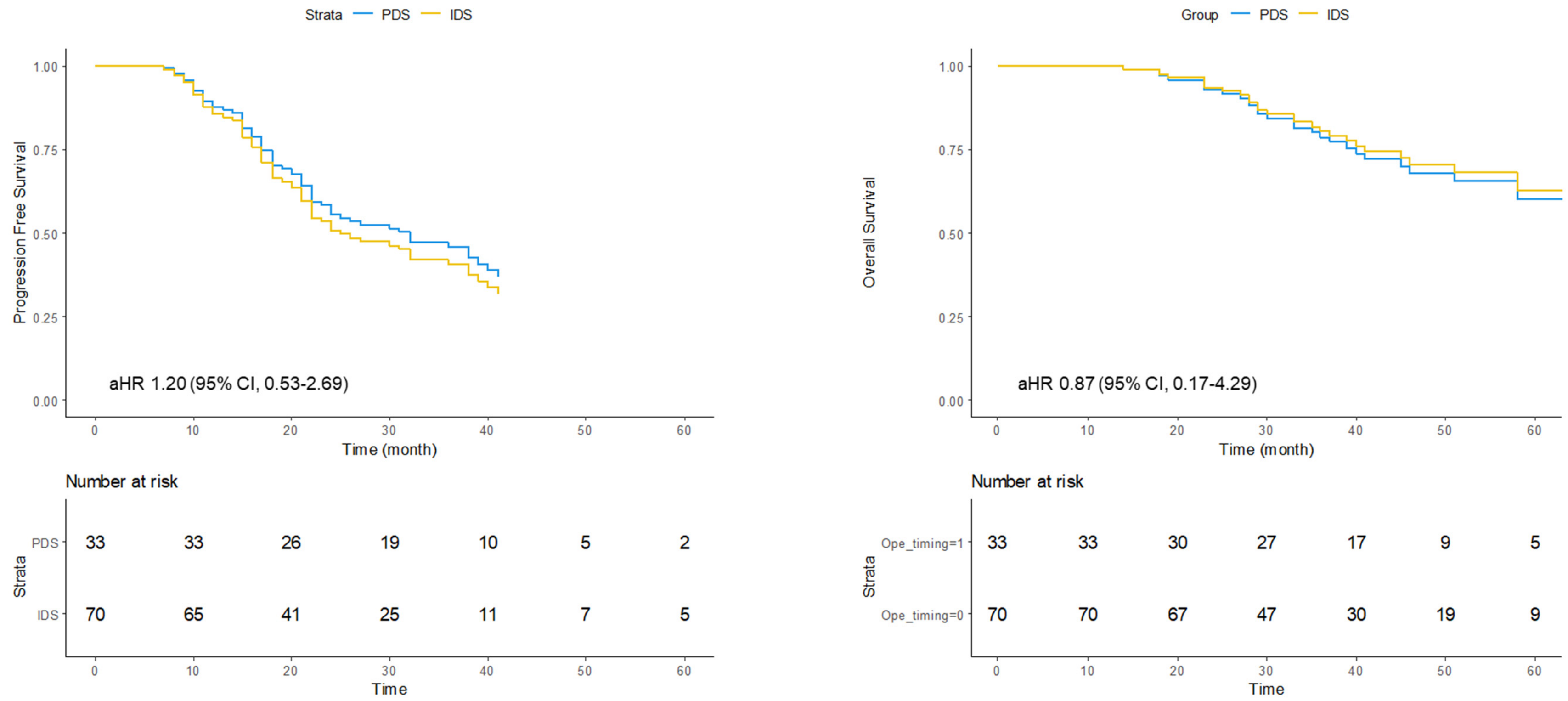

Figure S2. Adjusted survival curves for primary and interval debulking surgery among BRCA wild-type patients who achieved complete cytoreduction (R0). PDS, primary debulking surgery; IDS, interval debulking surgery; aHR, adjusted hazard ratio; CI, confidence interval; R0, no macroscopic residual disease.

https://doi.org/10.3390/cancers18020245

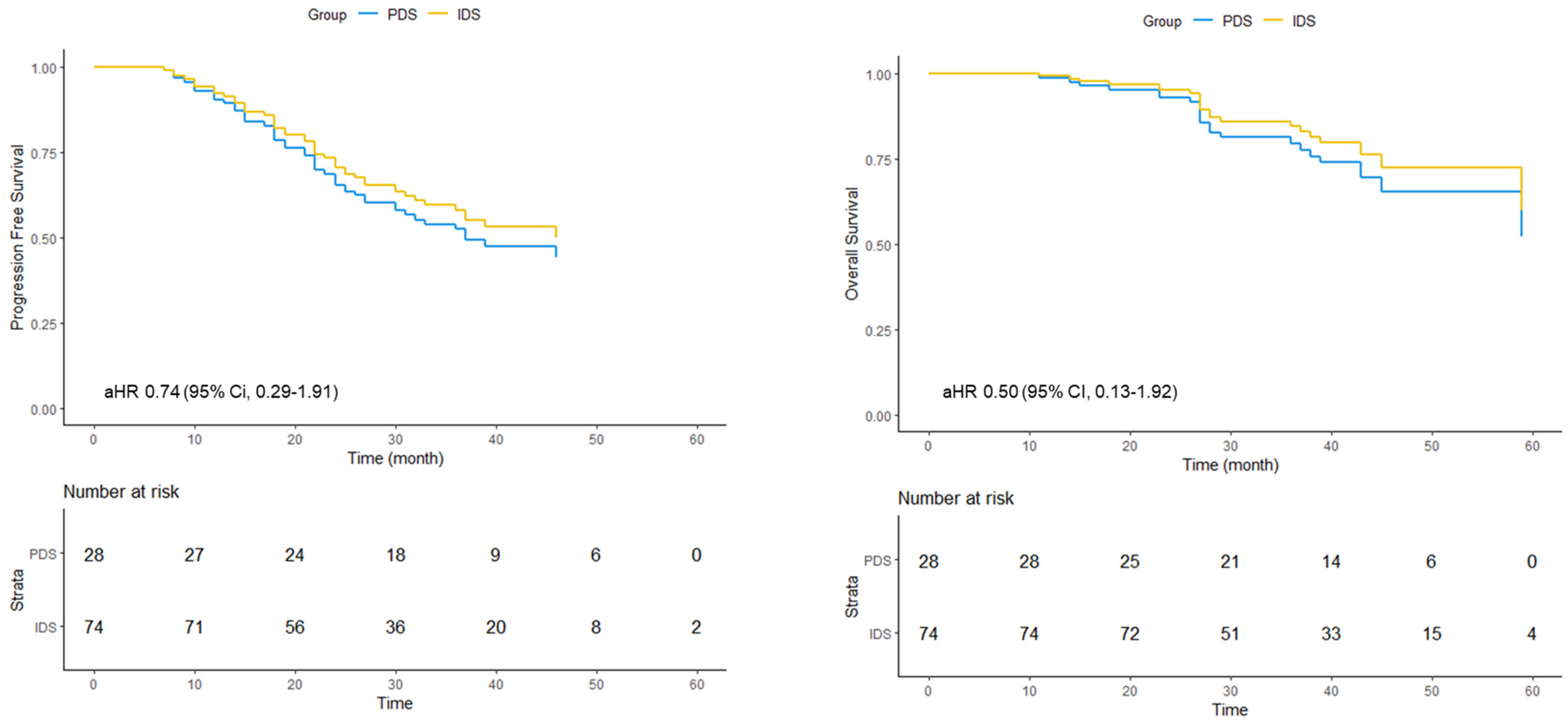

Figure S3. Adjusted survival curves for primary and interval debulking surgery among patients with homologous recombination–deficient (HRD) tumors. PDS, primary debulking surgery; IDS, interval debulking surgery; aHR, adjusted hazard ratio; CI, confidence interval.

https://doi.org/10.3390/cancers18020245
